# Activated α4 Integrins are Preferentially Expressed on Immature Thymocytes and Activated T Cells

**DOI:** 10.1080/1044667021000024229

**Published:** 2002-06

**Authors:** Fengyu Shu, Bernhard Holzmann, Frank Seibold, David Erle, John F. Kearney

**Affiliations:** 1 Division of Developmental and Clinical Immunology, Department of Microbiology University of Alabama at Birmingham 378 Wallace Tumor Institute 1824 6th Avenue South Birmingham AL 35294-3300 USA; 2 Institute for Medical Microbiology and Hygiene Technical University of Munich Munich D-81675 Germany; 3 Division of Gastroenterology, School of Medicine University of Alabama at Birmingham 378 Wallace Tumor Institute 1824 6th Avenue South Birmingham AL 35294-3300 USA; 4 Lung Biology Center Department of Medicine University of California at San Francisco San Francisco CA 94143 USA

## Abstract

We have identified a novel mAb, SG31, which recognizes the mouse integrin α4 subunit. Unlike the epitopes recognized by other anti-α4 antibodies, the SG31 epitope is expressed on subpopulations of thymocytes and peripheral T cells. After manganese ion, but not phorbol myristic acetate activation, the epitope is induced and expressed on the majority of peripheral T cells. These data suggest that the SG31 epitope is an activation epitope and that manganese ions activate α4 integrins by inducing a conformational change. Comparative flow cytometric analyses showed that the SG31 epitope as well as the epitope detected by other anti-α4 antibodies is expressed on all B lineage cells. In the T lineage, expression of the α4 integrins is down-regulated during thymocyte development. Although mature thymocytes still express the α4 integrins, they lose almost entirely the activation epitope recognized by SG31. In contrast, the most immature thymocytes express high levels of this epitope. In the periphery, SG31 epitope is expressed mostly by activated T cells, in contrast to the overall population of T cells that express the α4 integrins at homogenous levels. These results suggest that the activation of the α4 integrins is parallel to that of T cells.


*Developmental Immunology*, 2002 Vol. 9 (2), pp. 73–84

Taylor & Francis healthsciences

### Activated α4 Integrins are Preferentially Expressed on Immature Thymocytes and Activated T Cells

FENGYU SHU[a], BERNHARD HOLZMANN[b], FRANK SEIBOLD[c], DAVID ERLE[d] and JOHN F. KEARNEY[a,*]

[a]*Division of Developmental and Clinical Immunology, Department of Microbiology, University of Alabama at Birmingham, 378 Wallace Tumor Institute, 1824 6th Avenue South, Birmingham, AL 35294-3300, USA;* [b]*Institute for Medical Microbiology and Hygiene, Technical University of Munich, D-81675 Munich, Germany;* [c]*Division of Gastroenterology, School of Medicine, University of Alabama at Birmingham, 378 Wallace Tumor Institute, 1824 6th Avenue South, Birmingham, AL 35294-3300, USA;* [d]*Lung Biology Center, Department of Medicine, University of California at San Francisco, San Francisco, CA 94143, USA*

We have identified a novel mAb, SG31, which recognizes the mouse integrin α4 subunit. Unlike the epitopes recognized by other anti-α4 antibodies, the SG31 epitope is expressed on subpopulations of thymocytes and peripheral T cells. After manganese ion, but not phorbol myristic acetate activation, the epitope is induced and expressed on the majority of peripheral T cells. These data suggest that the SG31 epitope is an activation epitope and that manganese ions activate α4 integrins by inducing a conformational change. Comparative flow cytometric analyses showed that the SG31 epitope as well as the epitope detected by other anti-α4 antibodies is expressed on all B lineage cells. In the T lineage, expression of the α4 integrins is down-regulated during thymocyte development. Although mature thymocytes still express the α4 integrins, they lose almost entirely the activation epitope recognized by SG31. In contrast, the most immature thymocytes express high levels of this epitope. In the periphery, SG31 epitope is expressed mostly by activated T cells, in contrast to the overall population of T cells that express the α4 integrins at homogenous levels. These results suggest that the activation of the α4 integrins is parallel to that of T cells.

*Keywords*: α4 Integrins; Activation epitope; Monoclonal antibody; SG31

### INTRODUCTION

The α4 integrin α4β7 and α4β1 are heterodimeric cell surface adhesion molecules involved in leukocyte cell–cell and cell–matrix interactions and migration (Elices, 1994). α4β1 is distributed on essentially all hemopoietic lineage precursors and most mature blood cells except erythrocytes, platelets and neutrophils (Elices, 1994; Lobb and Hemler, 1994). α4β1 expression can be induced on activated neutrophils (Reinhardt *et al.*, 1996) and upregulated during T and B cell activation (Hemler, 1990; Postigo *et al.*, 1991). It is also expressed on non-lymphoid tissues in developing embryos (Stepp *et al.*, 1994; Sheppard *et al.*, 1994) and various non-hematopoietic tumor cells (Lobb and Hemler, 1994). The ligands for α4β1 are the alternatively spliced connecting segment-1 (CS1) region of extracellular matrix protein fibronectin and domains 1 and 4 of vascular cell adhesion molecule-1 (VCAM-1, CD106) (Elices, 1994). After its activation, α4β1 can also interact with other sequences on fibronectin, thrombospondin and the bacterial coat protein invasin (Lobb and Hemler, 1994). α4β7 is expressed on most lymph node T and B cells, on the gut homing subset of $CD4^+$ memory T cells, and on lymphocytes present in rheumatoid synovium (Lobb and Hemler, 1994). In human, natural killer (NK) cells, eosinophils, and newborn blood B and T cells show relatively homogeneous expression of α4β7, while adult blood B and T cells show more heterogeneous expression (Erle *et al.*, 1994). The known ligands for α4β7 include the fibronectin CS-1 region, VCAM-1, and the mucosal addressin cell adhesion molecule-1 (MAdCAM-1) (Lobb and Hemler, 1994). In addition, the α4 subunit itself may function as a ligand for α4β1 and α4β7 (Altevogt *et al.*, 1995).

The α4 integrins play important roles in embryogenesis (Stepp *et al.*, 1994) including lympho-hemopoiesis (Miyake *et al.*, 1991; Hamamura *et al.*, 1996), lymphocyte trafficking/homing (Strauch *et al.*, 1995; Abitorabi *et al.*, 1996), leukocyte extravasation during inflammation (van Dinther-Janssen *et al.*, 1991; Engelhardt *et al.*, 1995), lymphocyte activation during immune responses (Ferguson and Kupper, 1993), differentiation and generation of memory cells within the microenvironment of

*Corresponding author. Address: University of Alabama at Birmingham, USA. Tel.: +1-205-934-3370. Fax: +1-205-934-1875. E-mail:john.kearney@ccc.uab.edu

ISSN 1044-6672 print © 2002 Taylor & Francis Ltd
DOI: 10.1080/1044667021000024229

lymphoid germinal centers (Freedman *et al.*, 1990; Koopman *et al.*, 1991). The α4 deficient mice have defects in placental and cardiac development leading to fetal death (Yang *et al.*, 1995). In mice chimeric for α4 expression, T and B cell development is severely impaired in adults. In addition, T cell homing to Peyer's patches is blocked (Arroyo *et al.*, 1996). However, monocytes and NK cells can develop normally. α4β7 is involved in lymphocyte homing to intestinal mucosa (Cepek *et al.*, 1993; Strauch *et al.*, 1995). The other member of the β7 integrin subfamily, αEβ7, is expressed on intraepithelial lymphocytes (Kilshaw and Murant, 1990) and interacts with E-cadherin on intestinal epithelium (Cepek *et al.*, 1994). α4 Integrins are also involved in pathogenesis of metastatic melanoma, atherosclerotic plaques, and rheumatoid arthritis and disease progression in many animal models such as experimental autoimmune encephalomyelitis, autoimmune diabetes, antigen-induced asthma, and so on (Elices, 1994; Lobb and Hemler, 1994).

Use of monoclonal antibodies has contributed greatly to our current knowledge of α4 integrin structure and their functions. Here we describe the identification of a new anti-murine α4 mAb SG31. Comparative studies with SG31 and several other anti-mouse α4 mAbs revealed that SG31 recognizes an epitope which is not present on all α4 integrin molecules. This epitope can be up-regulated on peripheral T cells by manganese ions which reportedly activate α4 integrins by inducing conformational changes. Our data strongly suggest that SG31 recognizes an activation epitope and that α4 integrins exist in different activation statuses on B and T lymphocytes. SG31 may therefore provide an important tool to elucidate the mechanisms of α4 integrin-mediated physiological and pathological processes.

### RESULTS

### SG31 Recognizes the Integrin A4 Subunit

Preliminary immunofluorescence staining and flow cytometric analyses showed that mAb SG31 (rat γ2a, κ) reacts with blood cells of multiple lineages and it precipitated two subunits (160 and 100 kDa, respectively) from pro-B lymphoma cell line 38B9. Since integrins are the only known leukocyte antigens consisting of heterodimers with similar molecular weight, our initial data suggest that SG31 may react with α4β1 or αLβ2 because of their comparable distribution on leukocytes. When mouse bone marrow cells were co-stained with SG31 and anti-α4 or anti-αL antibodies, SG31 showed a staining profile similar to that of anti-α4, but not that of anti-αL (data not shown).

Since the integrin α4 subunit can associate with either β1 or β7 to form α4β1 and α4β7, we next attempted to determine the nature of the integrin subunit recognized by SG31. The cell lines 38B9 and TK-1, which express α4β1 and α4β7, respectively, were stained with SG31 and antibodies to α4 (PS/2), β1 (KMI6) and β7 (M293). We found that SG31 reacted with both cell lines irrespective of the associated β subunit (Fig. 1a). The data strongly suggested that SG31 recognizes α4 itself.

We reported previously that the α4 subunits on TK1 cells undergo post-translational cleavage giving rise to the $\alpha4^{80}$ and $\alpha4^{70}$ molecules, and that the association of α4 and β7 is dependent on the presence of cations (Ruegg *et al.*, 1992). To confirm that SG31 recognizes the α4 subunit, we did immunoprecipitation studies with TK1 using SG31 and other antibodies. In the presence of cations ($Ca^{2+}$, $Mg^{2+}$ and $Mn^{2+}$), SG31 precipitated proteins (lane 2) of similar size to those of $\alpha4^{150}$, β7, $\alpha4^{80}$ and $\alpha4^{70}$ as precipitated by PS/2 (lane 3) and M293 (lane 4) (Fig. 1b). In the absence of cations (depleted by EDTA and EGTA), SG31 precipitates only proteins (lane 7) of similar size to $\alpha4^{150}$ and $\alpha4^{80}$ as that precipitated by PS/2 (lane 8), M293 precipitated only β7 (lane 9). The control antibody (lanes 1 and 6) and KMI6 (anti-integrin β1 subunit) (lanes 5 and 10) did not precipitate any protein. That the same precipitating pattern is shared by SG31 and PS/2 strongly suggests that SG31 recognizes α4 subunits.

We have further confirmed that SG31 recognizes α4 by immunodepletion studies with 38B9 cells which express only α4β1 (Fig. 1c). Iodinated 38B9 cell lysates were depleted with PS/2- or SG31-coupled Affi-gel 10 beads. The depleted lysates were then precipitated with PS/2- or SG31-coupled beads, respectively (Fig. 1c). The undepleted lysates were precipitated with control antibody-, SG31-, or PS/2-coupled beads for comparison. SG31 precipitated proteins (lane 6) of similar size to α4 and β1 as precipitated by PS/2 (lane 7). It is noticeable that the amount of proteins precipitated by SG31 is smaller than that precipitated by PS/2. After PS/2 or SG31 depletion, the amount of proteins left which can be further precipitated by either PS/2 or SG31 is significantly reduced (lanes 1–4). The depletion was most dramatic when the lysates were precleared by PS/2 (lanes 1 and 2) and when the lysates were precleared with SG31 and then precipitated with SG31 (lane 4). After SG31 immunodepletion, there was still a significant amount of material remaining, which can be precipitated by PS/2 (lane 3). The control antibody did not precipitate any protein corresponding to α4 or β1 (lane 5). These data demonstrated that SG31 recognizes the murine integrin α4 subunits, more specifically the N-terminal fragment of α4 subunit, $\alpha4^{80}$, as does PS/2 (Fig. 1b). It was of interest that the α4 subunits on 38B9 cells do not undergo obvious cleavage in contrast to those on TK1 cells (Fig. 1b,c).

### SG31 Recognizes an Epitope Present only on a T Cell Population

Our previous studies have shown that SG31 staining is less intense than that of PS/2 on some cell lines (data not shown) and SG31 precipitated smaller amount of α4 integrin molecules than PS/2 (Fig. 1). These

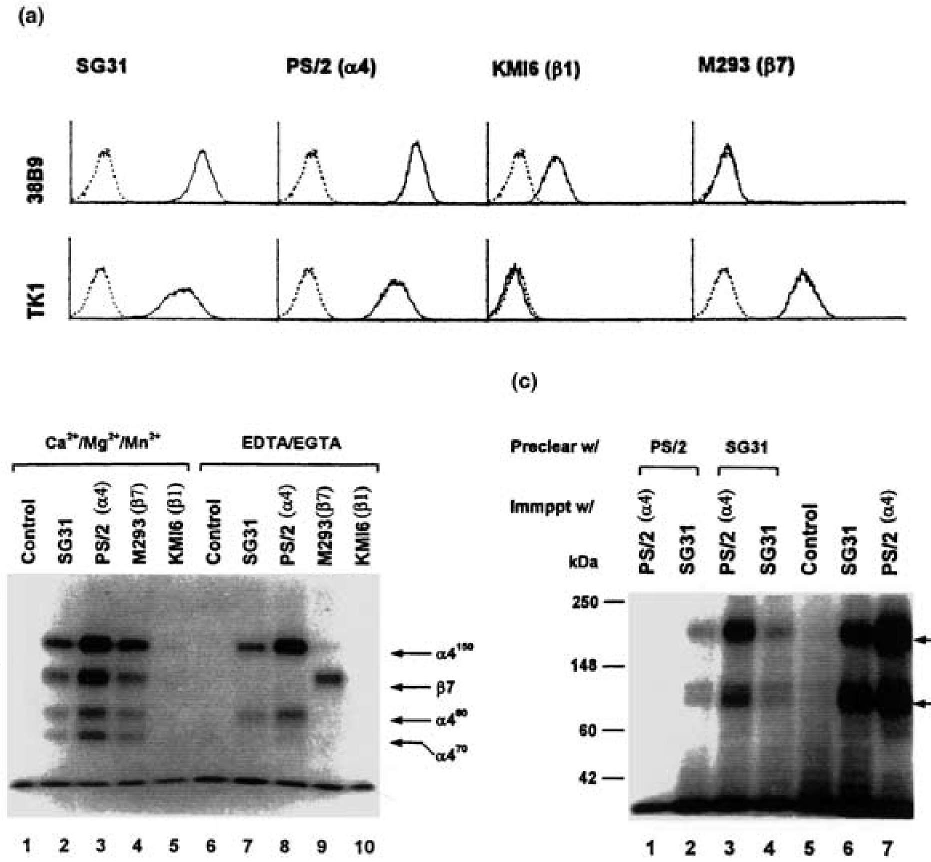

FIGURE 1 SG31 recognizes the integrin α4 subunit. (a) SG31 reacted with α4-expressing cell lines no matter what β subunit is expressed. 38B9 and TK1 cell lines were stained with control antibody (dotted line), SG31, PS/2 (anti-α4), KMI-6 (anti-β1), and M293 (anti-β7). Stained cells were analyzed by flow cytometry. (b) Immunoprecipitation patterns of TK1 cells by SG31 and PS/2 are the same. TK1 lymphoma cells were iodinated and lysed with 1% NP-40 lysis buffer supplemented with cations or depleted of cations. Precleared lysates were incubated with a control Ab-, SG31-, PS/2-, m293-, and KMI6-coupled beads and run under non-reducing conditions. Similar results were obtained under reducing conditions (data not shown). (c) SG31 and PS/2 depleted the integrin α4 and β1 subunits from 38B9 cells. Precleared lysates were further depleted with 3 rounds of PS/2- or SG31-coupled Affi-gel 10 beads and precipitated with PS/2- and SG31-coupled Affi-gel 10 beads. Lysates without depletion were precipitated with control Ab-, SG31-, and PS/2-coupled beads for comparison.

findings suggest that SG31 recognizes an epitope that is not present on all α4 integrin molecules. To test this possibility, we stained freshly harvested spleen and mesenteric lymph node cells of young BALB/c mice and compared the staining intensities of SG31 and PS/2 on B ($IgM^+$) and T ($CD3^+$) cells (Fig. 2). Our data revealed that SG31 stained most $IgM^+$ cells in spleen and mesenteric lymph node as did PS/2, though SG31 staining is more heterogeneous than that of PS/2. Interestingly, SG31 stained only a minor subpopulation of $CD3^+$ cells in both tissues while PS/2 stained almost all T cells. In spleen, both SG31 and PS/2 stained brightly a $IgM^-CD3^-$ population which is presumably of myeloid lineage.

### All B Lineage Cells Express the SG31 Epitope

In order to further characterize the expression of the SG31 epitope in different lymphocyte compartments, we compared the staining of SG31 and PS/2 on different lymphocyte subsets using three-color staining and flow cytometric analyses. Bone marrow is the primary hemopoietic organ in adult mice and generates all lineages of blood cells except T cells. We focused our studies on B lineage cells in the bone marrow and stained bone marrow cells from young adult BALB/c mice with control antibody, SG31, or PS/2 (detected by PE-conjugated goat anti-rat IgG), CY2-conjugated anti-B220, and FITC-conjugated anti-CD43 or anti-IgM. B lineage cells in the bone marrow can be delineated into sequential stages based on the expression of surface markers ($B220^+CD43^+$ pro-B cells, $B220^+CD43^-IgM^-$ pre-B cells, $B220^+IgM^+$ B cells) (Hardy *et al.*, 1991). Our studies showed that all B lineage cells in each compartment of the bone marrow express the α4 integrins and the SG31 epitope (Fig. 3a and b).

Splenic B cells can be divided into three subsets based on surface expression of IgM and CD21 by flow cytometric analysis. Follicular (FO) B cells are $IgM^+CD21^+$, marginal zone (MZ) B cells $IgM^{high}$ $CD21^{high}$, and newly-formed (NF) B cells $IgM^{high}$ $CD21^{low}$ (Kraal, 1992; Oliver *et al.*, 1997). Spleen cells from young adult BALB/c mice were stained with control

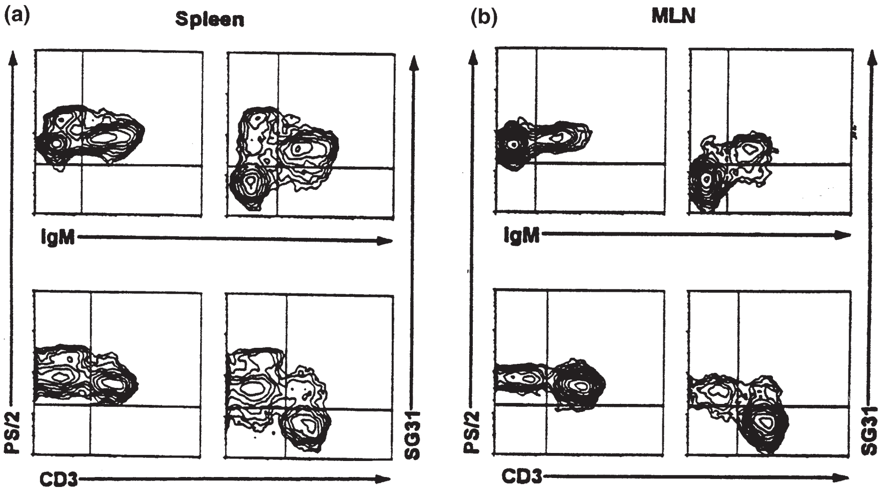

FIGURE 2 SG31 epitope is not present on all α4 integrin molecules, especially on T cells. Spleen or mesenteric lymph node cells from young BALB/c mice were stained with PS/2 or SG31 and anti-IgM or anti-CD3, and analyzed with flow cytometry.

antibody, SG31, or PS/2 (detected by PE-conjugated goat anti-rat IgG), FITC-conjugated anti-IgM, and biotinylated anti-CD21 (detected by SA-CU5). Flow cytometric analysis showed that all three B cell subsets express the α4 integrins and the SG31 epitope (Fig. 3c). However, SG31 staining again seems more heterogeneous than that of PS/2 (Fig. 3a–c).In summary, detailed analyses of the early B cell compartments and peripheral B cell subsets showed no major difference in expression of the SG31 epitope and total α4 integrins as detected by PS/2.

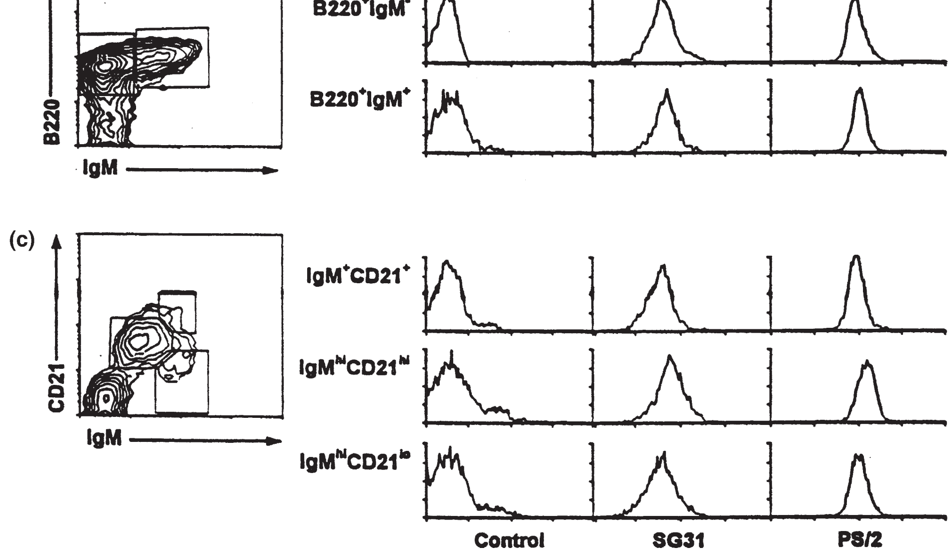

FIGURE 3 All B lineage cells express the SG31 epitope. Bone marrow cells gated from the different compartments in young adult BALB/c mice were all stained with SG31 and PS/2.

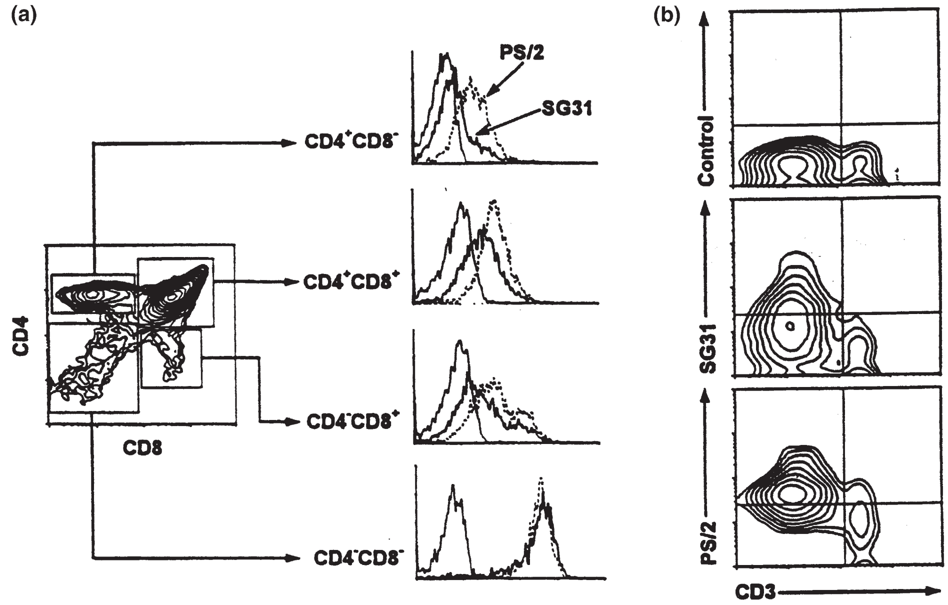

FIGURE 4 CD4$^-$CD8$^-$ thymocytes express high levels of the SG31 epitope. (a) Subpopulations of thymocytes from young adult BALB/c mice expressing SG31 (thick solid line), and PS/2 (broken line). Expression of the SG31 and PS/2 epitopes was compared on thymocyte subpopulations based on CD4, CD8 expression. (b) Thymocytes were stained with FITC-conjugated anti-CD3, and control antibody, SG31, or PS/2, detected by PE-conjugated goat anti-rat IgG.

### CD4$^-$CD8$^-$ Thymocytes Express High Levels of the SG31 Epitope

We have observed that SG31 reacted with fewer thymocytes than PS/2 (see below, Fig. 6). Then we tested the expression of the SG31 epitope at different stages of thymocyte development. Our data showed that CD4$^-$CD8$^-$ (DN) thymocytes express the α4 integrins at high levels as detected by anti-integrin α4 mAb PS/2. During maturation, their expression is down-regulated and CD4$^+$ or CD8$^+$ (SP) thymocytes express the α4 integrins at low levels as reported {94}. The SG31 epitope is expressed at the same high levels as the PS/2 epitope on DN thymocytes, however, its expression is down-regulated to a larger extent on more mature CD4$^+$CD8$^+$ (DP) and SP thymocytes (Fig. 4a). A subset of CD4$^-$CD8$^+$ thymocytes express the α4 integrins and the SG31 epitope at high levels. These cells are most likely at the transitional stage from DN to DP, since α4 integrins are expressed at low levels by only a minor subpopulation of CD3$^+$ thymocytes and the SG31 epitope is not expressed by CD3$^+$ thymocytes at all (Fig. 4b).

### Activated T Cells Express Higher Levels of α4 Integrins and Express the SG31 Epitope

To determine if only activated T cells express the SG31 epitope, we stained splenic and mesenteric lymph node cells with SG31 together with antibodies against other differentiation/activation antigens. Activated T cells express CD69 and high levels of CD44. We found that only CD69$^+$ and CD44$^{high}$ T cells express the SG31 epitope. While almost all peripheral T cells express α4 integrins, CD69$^+$ and CD44$^{high}$ T cells express higher levels of these integrins as detected by PS/2 (Fig. 5a). Activated T cells also up-regulate surface expression of CD11a, CD25, CD38, and CD54 (Brines, 1997). They may down-regulate surface expression of CD62L. The expression of these activation markers together with CD95 was compared between SG31$^+$ (solid line) and SG31$^-$ (broken line) mesenteric lymph node T cells (Fig. 5b). Flow cytometric analyses showed that SG31$^+$ T cells express higher levels of CD11a and CD54. SG31$^+$ T cells are also enriched for CD62L$^-$, CD25$^+$, and especially CD38$^+$ cells. Neither SG31$^+$ nor SG31$^-$ T cells express significant amount of CD95. Taking all these results together, SG31$^+$ T cells appear to express a constellation of markers typical of activated T cells.

### Cells that Express High Levels of α4 Integrins also Express High Levels of the SG31 Epitope

Since the activity of integrins can be modulated at several levels including cell surface expression and affinity maturation (Garratt and Humphries, 1995), we next compared the expression of α4 integrins and the SG31 epitope in peritoneal cavity, bone marrow, peripheral blood, splenic, mesenteric lymph node, Peyer's patch cells and thymocytes from young adult BALB/c mice by staining the cells with SG31 and the FITC-conjugated anti-α4 mAb, 9C10. 9C10 is a commercially available Ab which recognizes α4 integrins. We have previously shown that SG31 and 9C10 do not block each other in terms of binding to TK1 cells and 9C10, PS/2 and R1-2 show similar staining patterns on fetal and adult lymphoid tissues which differ from that of SG31 (data not shown).

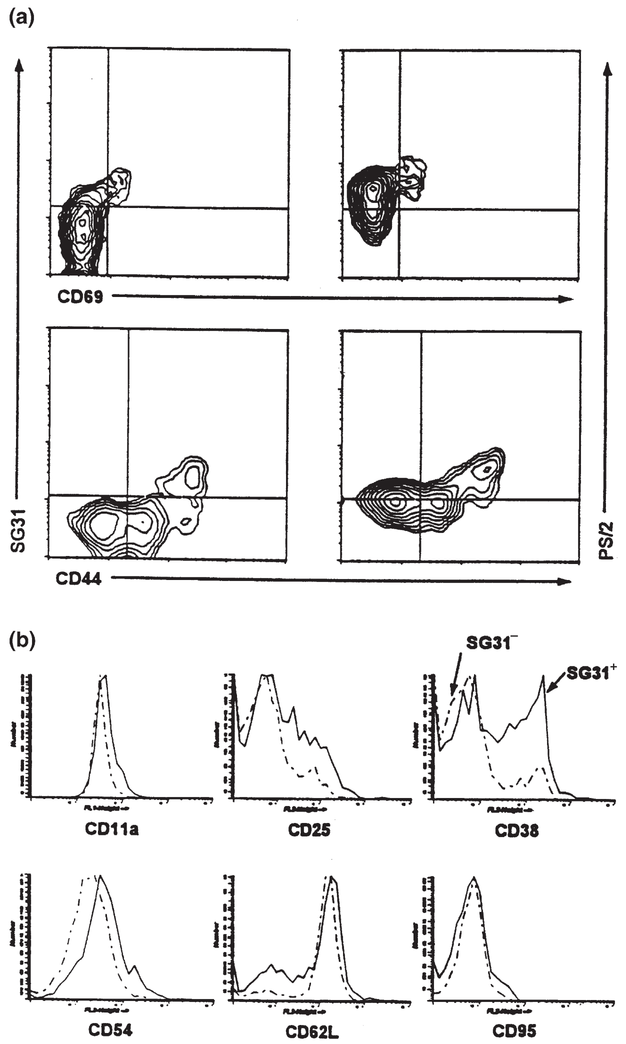

FIGURE 5 Activated T cells express activated α4 integrins. (a) Only $CD69^+$ and $CD44^{high}$ spleen cells express the SG31 epitope. (b) Staining of mesenteric lymph node cells showed that $SG31^+$ T cells express higher levels of CD11a and CD54. $SG31^+$ T cells are also enriched for $CD62L^-$, $CD25^+$ and especially $CD38^+$ cells. Neither $SG31^+$ nor $SG31^-$ T cells express CD95.

The reason that we did not stain cells with SG31 and PS/2 or R1-2 is that these Abs blocked the binding of each other. Flow cytometric analysis showed that both SG31 and 9C10 reacted with all lymphocytes in peritoneal cavity (Fig. 6). 9C10 stained most cells in the lymphocyte gate in all other tissues. SG31 stained the cells which were stained brightly by 9C10, however, it did not react with a significant number of cells which were stained modestly by 9C10 in most lymphoid tissues, especially thymus (consistent with Fig. 4). These results showed that the cells expressing high levels of the α4 integrins also express high levels of the SG31 epitope, while the cells expressing moderate levels of α4 integrins may not express or express only low levels of the SG31 epitope.

### SG31 Epitope is Up-regulated by $Mn^{2+}$ on T Cells

It has been reported that TK1 cells use α4β7 to bind MAdCAM-1 under flow conditions. Lymph node cells can bind efficiently under similar test conditions only after their activation by PMA or $Mn^{2+}$ (Bargatze *et al.*, 1995; Berlin *et al.*, 1995). SG31 and PS/2 have similar staining intensity on TK1 cells (Fig. 1a), but they have very different reactivity on peripheral T cells. These differences led us to speculate that SG31 may recognize the activated form of α4 integrins. To test this hypothesis, we first treated splenic and mesenteric lymph node cells with either $CaCl_2$, $MgCl_2$, or $MnCl_2$ at 2 mM (the concentration widely used in the literature), then stained with control antibody, SG31 or PS/2 together with FITC-conjugated anti-CD5. We found that after $Mn^{2+}$-activation, the expression of SG31 epitope was up-regulated on splenic (data not shown) and lymph node T cells (Fig. 7). $SG31^+$ T cells increase from 10 to 20% in spleen and from 13 to 40% in mesenteric lymph node after $Mn^{2+}$-activation. The staining of B cells was not affected. $Mn^{2+}$-activation did not change PS/2 staining on either B or T cells. $Ca^{2+}$ and $Mg^{2+}$ at the same concentration (2 mM) do not have significant effect on SG31 epitope induction on T cells, consistent with their apparent failure to activate α4 integrins. PMA can also activate α4 integrins on T cells as previously reported (Bargatze *et al.*, 1995). We found that PMA stimulation (for 3 days) induced expression of CD69 and CD25, up-regulated CD44 expression on subsets of T cells, and significantly increased the number of T cells expressing α4 integrins but not the SG31 epitope (data not shown). However, not all $CD69^+$ or $CD25^+$ T cells express the SG31 epitope. Similarly, not all $SG31^+$ cells express CD69 or CD25, again consistent with the findings with fresh tissues in that $SG31^+$ T cells are enriched for, but not exclusively, activated cells (Fig. 5b). Therefore, there is no simple relationship between T cell activation and α4 integrin activation.

### SG31 Selectively Inhibits 38-β7 Cell Adhesion to Fibronectin

38-β7 Cell line is a 38C13 pre-B lymphoma transfected with murine β7 cDNA. Therefore, it expresses α4β7 and can adhere to fibronectin, VCAM-1 and MAdCAM-1. We tested SG31 for its ability to inhibit 38-β7 cell adhesion to these ligands. We found that PS/2 antibody inhibited 38-β7 cell adhesion to all these three ligands, whereas SG31 inhibited only the adhesion of 38-β7 cells to fibronectin, but not CHO cells expressing VCAM-1 or MAdCAM-1 (Fig. 8).

### DISCUSSION

The major findings of this report are that: (i) mAb SG31 recognizes the mouse integrin α4 subunit, (ii) most T cells express α4 integrins but not the SG31 epitope, which is in contrast to B cells all of which express the SG31 epitope, (iii) SG31 epitope is up-regulated on T cells by $Mn^{++}$, (iv) primarily immature thymocytes and activated T cells express SG31 epitope, (v) PMA-activated splenic T cells

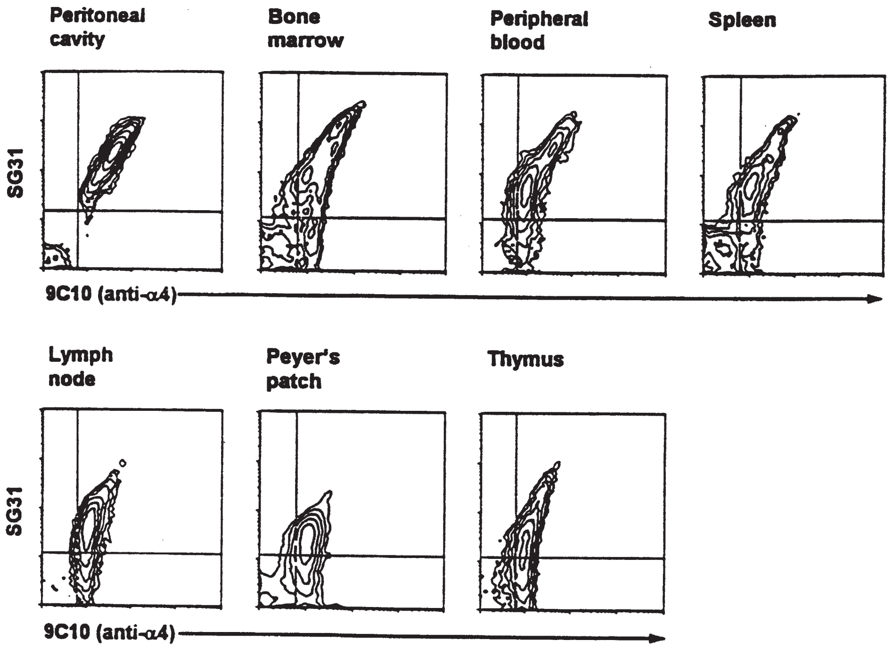

FIGURE 6 Cells expressing high levels of α4 integrins express them as active forms. Peritoneal cavity, bone marrow, peripheral blood, splenic, mesenteric lymph node, Peyer's patch cells and thymocytes from young adult BALB/c mice were stained with SG31 and 9C10 (anti-α4), and analyzed by flow cytometry.

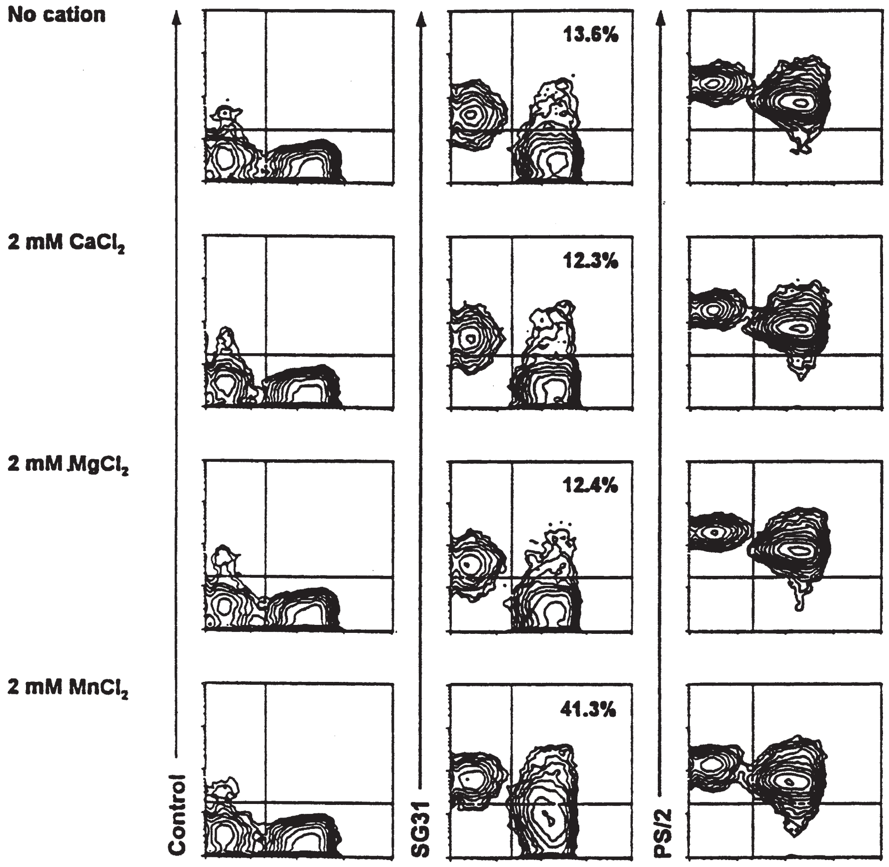

FIGURE 7 SG31 recognizes an activation epitope that is up-regulated by $Mn^{2+}$.

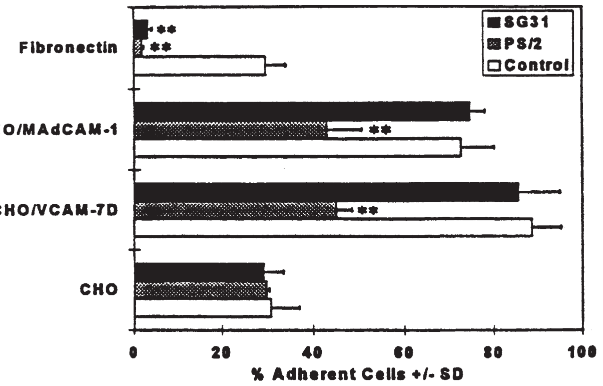

FIGURE 8 SG31 selectively inhibits 38-β7 cell adhesion to fibronectin. The data are represented as mean and standard deviation from three independent experiments. Statistical differences between adhesion in the presence of control ab and SG31 or PS/2 were determined by Student's *t* test ($^{**}p < 0.001$).

up-regulate α4 integrins and SG31 epitope (data not shown), and (vi) cells expressing high levels of α4 integrins also express high levels of SG31 epitope.

### SG31 Recognizes Activated Murine α4 Integrins

Immunoprecipitation and immunodepletion studies clearly demonstrated that SG31 recognizes the mouse integrin α4 subunit, and more specifically, the $\alpha4^{80}$ fragment. Flow cytometric and immunoprecipitation data also revealed that mAb SG31 does not react with all α4 integrin molecules. These finding together with the observation that the SG31 epitope is up-regulated on T cells by $Mn^{2+}$ strongly suggests that this antibody recognizes an activation epitope on α4 integrin molecules.

It is known that the activity of α4 integrins can be up-regulated by $Mn^{2+}$ and PMA without an overall increase of expression on the given cells (Garratt and Humphries, 1995). Accumulating data suggest that $Mn^{2+}$ and PMA activate α4 integrins by different mechanisms. $Mn^{2+}$, like $Ca^{2+}$, $Mg^{2+}$, and some mAbs, may induce conformational changes by direct interactions with the integrin molecule (Ginsberg *et al.*, 1992). Shimizu and Mobley reported that cell activation with PMA treatment can further enhance T cell adhesion to fibronectin and VCAM-1 in the presence of $Mn^{2+}$ (Shimizu and Mobley, 1993). Since PMA is a protein kinase C activator, phosphorylation of integrin cytoplasmic domains or integrin associated proteins may play a role in integrin activation. Studies on other members of the integrin family, such as αLβ2 and αVβ5, showed that PMA increases adhesion by cytoskeletal-dependent processes such as mobilization and cluster formation of integrin molecules, and cell spreading, but not by affinity modulation (Stewart *et al.*, 1996). Identification of activation epitopes on human β1 and β2 integrins has been reported (Picker *et al.*, 1993; Bazzoni *et al.*, 1995). In particular, the activation epitope of β2 integrins, especially αLβ2, recognized by mAb 24 was well studied (Dransfield *et al.*, 1992; Picker *et al.*, 1993). Our data identify for the first time an activation epitope on the integrin α4 subunit. Unlike the mAb 24 epitope, the SG31 epitope is present on a subpopulation of peripheral T cells in the absence of cations (when depleted by EDTA, a chelator for all bivalent cations). This activation epitope is significantly induced by $Mn^{2+}$ on splenic and lymph node T cells. Similar to the mAb 24 epitope, but to a much less extent, $Ca^{2+}$ down-regulates and $Mg^{2+}$ (especially when $Ca^{2+}$ ions were depleted by EGTA, a $Ca^{2+}$-specific chelator) up-regulates the SG31 epitope (data not shown). We observed that short-period stimulation with PMA (30 min) does not up-regulate the SG31 epitope (data not shown), while long-period stimulation (3 days) up-regulate activation markers such as CD44, CD69, CD25, and the SG31 epitope. These data confirm that $Mn^{2+}$ and PMA activate α4 integrins by different mechanisms. The up-regulation of the SG31 epitope by $Mn^{2+}$ is dependent on the presence of $Mn^{2+}$ during the subsequent staining procedure, indicating that $Mn^{2+}$ induces conformational change of α4 integrins by binding the molecules. Our finding is consistent with the previous report that the effects of $Mn^{2+}$-activation wears off shortly after $Mn^{2+}$ depletion (Bargatze *et al.*, 1995). The effect of $Mn^{2+}$ on SG31 epitope induction is maximal at a concentration of 2 mM which is widely reported in the literature. Higher concentration of $Mn^{2+}$ caused significant cell damage. $Mn^{2+}$ at 2 mM is likely to be an artificial stimulus whose physiological equivalent is still unknown.

α4 integrins can exist in a range of activation states (Masumoto and Hemler, 1993; Lobb and Hemler, 1994). A recent report provides evidence that cells may express three populations of integrins: inactive (not responsive to activating signals), transiently active or low affinity (responsive to ligand and activating signals), and stably active and high affinity (readily occupied by ligand) (Yednock *et al.*, 1995). $Mn^{2+}$-activation does not induce the SG31 epitope on all peripheral T cells (Fig. 7). One possible explanation is that the $Mn^{2+}$-responsive α4 integrins represent the second population mentioned above. The up-regulation of the SG31 epitope on T cells was not due to the binding of soluble ligands because the contents of the buffers used in our experiments were well defined. In other experiments, buffer containing fetal calf serum, a source of significant amount of fibronectin, was used and no significant difference was observed in terms of the expression of SG31 epitope.

At least three independent mechanisms are utilized to increase adhesive activities of the α4 integrins: (i) increased levels of expression, (ii) clustering of α4 integrin receptors and (iii) conformational change and activation by extracellular and intracellular signals (Garratt and Humphries, 1995). Lymphoid cells expressing high levels of the α4 integrins as detected by PS/2 also express high levels of SG31 epitope, while cells expressing moderate levels of the α4 integrins may not express this epitope at all (Fig. 6). Our data demonstrate that lymphocytes maximize their adhesive activities by expressing higher levels of α4 integrins as active forms.

### Different Usage of α4 Integrins on B and T Lymphoid Lineages

Although B cells have a need to regulate their adhesion activities during development, out data show that almost all B lineage cells express α4 integrins and the SG31 epitope independent of their stage of development (Fig. 3). In addition, the SG31 epitope on B cells is not further up-regulated by $Mn^{2+}$, suggesting that the α4 integrins on B cells are constantly active and can be readily occupied by ligands. Thus, other adhesion molecules on B lineage cells or ligands of α4 integrins on stromal and endothelial cells may be critical for controlling the migratory behaviors of B cells.

In contrast, expression levels and activation states of α4 integrins seem to play essential roles in thymocyte development and activated T cell migration. The expression of α4 integrins as detected by PS/2 is the highest on the most immature DN thymocytes, and then it is down-regulated with further thymocyte maturation. The α4 integrins are expressed at low levels on mature SP thymocytes (Fig. 4). The expression of the SG31 epitope does not always parallel that of the overall levels of α4 integrins. DN thymocytes express the SG31 epitope at the same high levels as that of α4 integrins (Fig. 4). SP thymocytes are almost negative for the SG31 epitope though they still express low levels of α4 integrins. This pattern of expression suggests the important roles of α4 integrins in thymocyte–stromal interactions at early stages of development. In peripheral lymphoid tissues, all T cells express the α4 integrins as detected by PS/2, but only small subpopulations (both $CD4^+$ and $CD8^+$, data not shown) express the SG31 epitope (Fig. 2). Compared to $SG31^-$ T cells, $SG31^+$ T cells express CD69, higher levels of CD44, CD11a and CD54 (Fig. 5a and B). The $SG31^+$ population is also enriched for $CD62L^-$, $CD25^+$, and especially $CD38^+$ cells in peripheral lymphoid tissues, such as mesenteric lymph nodes. These results suggest that $SG31^+$ T cells may be predominantly, activated T cells. Memory $CD4^+$ T cells are $CD11a^{high}$, $CD44^{high}$ and $CD45RB^{dull}$, and are heterogeneous in terms of homing receptor expression. They can be divided into $\alpha E\beta 7^{high}$, $\alpha 4\beta 7^{high}$ (further subdivided into $CD62L^+$ and $CD62L^-$) and $\alpha 4\beta 1^{high}$ subsets (Andrew *et al.*, 1996). Our data demonstrate that the activation epitope of α4 integrins as detected by SG31 can be a valuable addition to these activation markers. St-Pierre *et al.* recently demonstrated by adhesion assays that α4β1 is constitutively expressed in its high-avidity state during the early stages of T cell development (St-Pierre *et al.*, 1996). At later stages, mature thymocytes turn off α4β1 as well as αLβ2 functions. Only antigen-challenged peripheral mature T cells turn on the adhesion function of α4β1 and αLβ2. Our data fit with these observations and show that the expression of SG31 epitope reflects the fluctuation of α4β1 functions. Furthermore, our data demonstrate the different roles of α4 integrins in the development and function of B and T cell lineages.

### SG31 Provides a Unique Tool to Study α4 Integrins

Our results also confirmed that the association of α4 and β7 subunits on TK1 cells is dependent on the presence of cations (Ruegg *et al.*, 1992). $\alpha 4^{70}$ band was missing from the SG31 precipitates in the absence of cations. This observation suggests that β7 is important for maintaining the structure of α4 subunits, especially the cleaved ones. In other word, there is little interaction between $\alpha 4^{80}$ and $\alpha 4^{70}$. In contrast to α4 subunits on TK1 cells, the α4 subunits in association with β1 on 38B9 cells do not undergo significant cleavage (Fig. 1c), supporting that the cleavage of the integrin α4 subunits is cell-type specific.

Due to lack of crystallographic structural analysis of α4 integrins, current knowledge of their structure–function relationship is mainly derived from activity studies using mAbs and truncated, chimeric and site-mutated α4 constructs. Cross-inhibition, inhibition of ligand binding and induction or inhibition of homotypic cell aggregation studies using a large panel of anti-human α4 Abs identified three distinct and independent adhesion activities (Pulido *et al.*, 1991). Our preliminary studies suggest that SG31 recognizes a region close to the N-terminus of the α4 subunit. SG31 does not significantly induce TK1 cell aggregation, nor does it significantly inhibit TK1 cell aggregation induced by other mAbs. Adhesion studies showed that SG31 inhibits α4β7-expressing cell adhesion to fibronectin, but not to VCAM-1 or MAdCAM-1 (Fig. 8). Functional studies showed that SG31 did not affect lymphopoiesis when given *in vivo*. It did not affect thymocyte development in fetal thymic organ culture or B cell development in long-term bone marrow culture or bone marrow-stromal culture (data not shown). However, mice treated with SG31 had a dramatic reduction of $CD62L^+$ T cells in the peripheral blood (data not shown), suggesting that SG31 interfered with T cell trafficking. In addition, our data showed that most mesenteric lymph node T cells from mice with an inflammatory bowel disease (scid transferal model) express the SG31 epitope unlike their counterparts in normal mice (manuscript in preparation), in agreement with the previous findings that α4 integrins are involved in the pathogenesis of this group of diseases. These findings demonstrate that SG31 can be an important tool to examine the activation status of α4 integrins in animals with different diseases in which α4 integrins are involved, to study the structural basis of molecular interactions involved in the α4 integrins and their ligands, and to explore the physiological and pathological functions of such interactions.

### MATERIALS AND METHODS

### Animals

BALB/c and C3H/HeJ mice were purchased from Charles River Laboratories, Inc. (Wilmington, MA) and bred in the animal facility at the University of Alabama at Birmingham.

### Antibodies

Unless specified, antibodies were purchased from PharMingen, San Diego, CA. The following antibodies were used in this study: FITC-conjugated 145-2C11 (anti-CD3$\epsilon$), 53-7.313 (anti-CD5), 53-6.7 (anti-CD8), 2D7 (anti-integrin $\alpha$L subunit, CD11a), S7 (anti-CD43), 9C10 (anti-integrin $\alpha$4 subunit, CD49d), H1.2F3 (anti-VEA, CD69), polyclonal anti-mouse IgM, phycoerythrin (PE)-conjugated IM7 (anti-CD44), Jo2 (anti-Fas, CD95), and biotinylated-GK1.5 (anti-CD4), 7G6 (anti-CD21), 7D4 (anti-CD25), 90 (anti-CD38), 3E2 (anti-ICAM-1, CD54), and MEL-14 (anti-L-selectin, CD62L). Cy-Chrome (CY5)-conjugated 14.8 (anti-B220, CD45R) was purchased from Southern Biotechnology Associates, Inc. (Birmingham, AL). Hybridoma M293 (anti-integrin $\beta$7 subunit) was generously provided by PJ Kilshaw. Hybridoma PS/2 (anti-integrin $\alpha$4 subunit) and KMI6 (anti-integrin $\beta$1 subunit) were generously provided by P.W. Kincade. An irrelevant rat $\gamma$2a antibody (PharMingen) was used as isotype-matched control. Second step reagents were used as follows: FITC- or PE-conjugated goat anti-rat IgG (GIBCO-BRL, Life Technologies Corporate, Gaithersburg, MD) and Streptavidin (SA)-CY5 (Southern Biotechnology Associates, Inc., Birmingham, AL). Antibodies from culture supernatants were purified on protein G Sepharose (Pharmacia LKB, Uppsala, Sweden) and quantified by measuring optical density at 280 nm.

### Cell Lines

38B9 is a pro-B lymphoma expressing integrin subunits $\alpha$4 and $\beta$1. TK-1 is a spontaneous AKR/cum T cell lymphoma expressing $\alpha$4 and $\beta$7. 38-$\beta$7 cell line is a 38C13 pre-B lymphoma transfected with murine $\beta$7 cDNA. It expresses $\alpha 4\beta 7$, but not $\alpha 4\beta 1$

### Generation of Hybridoma SG31

A subpopulation of Ab8$^+$ bone marrow cells (Moratz *et al.*, 1994) was FACS sorted and used to immunize a rat subcutaneouly in multiple times at sites drained by the popliteal and paraaortic lymph nodes. Cells from the draining lymph nodes were then fused to the mouse plasmacytoma cell line Ag8.653 resulting in the isolation of Ab SG31 by screening against a variety of mouse lymphoid tissues and cell lines. Purified SG31 were used in this study and detected by PE- or FITC-conjugated goat anti-rat IgG.

### Flow Cytometry

Cells isolated from mouse lymphoid tissues or cell lines were incubated with purified mAb (20 μg/ml) in PBS containing 2% FCS and 0.01% sodium azide for 20 min on ice, washed in PBS containing 2% FCS and incubated with PE-conjugated goat anti-rat IgG for 20 min on ice. Flow cytometric analysis was performed on a FACScan or a FACSCalibur (Becton Dickinson, Mountain View, CA). The data were analyzed with WinList (Verity Software House, Inc., Topsham, ME) or WinMDI (public domain software, programmed by Joseph Trotter, obtained from Scripps Research Institute) on personal computers. For two- and three-parametric analysis, the cells were blocked by incubation with 20 ml of normal rat serum for 20 min on ice after the procedure described above to saturate the binding sites on goat anti-rat IgG and then stained with FITC-conjugated and biotinylated antibodies to other surface markers. Biotinylated antibodies was detected by SA-CY5.

To study the effects of $\alpha$4 integrin activation on SG31 epitope expression, mouse lymphocytes were activated with $Ca^{2+}$, $Mg^{2+}$ or $Mn^{2+}$ as described (Bargatze *et al.*, 1995; Berlin *et al.*, 1995). Briefly, splenic and mesenteric lymph node cells were incubated at room temperature in $Ca^{2+}/Mg^{2+}$-free Hanks balanced salt solution (HBSS) plus 10 mM HEPES (pH 7.2), pelleted, and resuspended in HBSS/HEPES (control cells) or HBSS/HEPES containing 2 mM $CaCl_2$, $MgCl_2$ or $MnCl_2$. The cells were stained accordingly and analyzed by flow cytometry. The same amount of cations were added in all reagents through the staining procedure.

### Immunoprecipitation

Radioiodination of TK-1 surface molecules was performed by the lactoperoxidase method as described by Laemmli (1970). The labeled cells were solubilized on ice for 30 min in lysis buffer (50 mM Tris–HCl [pH 7.5], 150 mM NaCl, 5 mM EDTA, 20 mM iodoacetamide, 0.1% sodium azide, aprotonin [2 mg/ml], 1 mM PMSF, soybean trypsin inhibitor [100 μg/ml], leupeptin [1 μg/ml], 20 mM $\epsilon$-amino-*n*-caproic acid, antipain [2 μg/ml], chymostatin [100 μg/ml], pepstatin [1 μg/ml], supplemented with 1% NP-40. The lysis buffers were either supplemented with cations by adding 5 mM $CaCl_2$, $MgCl_2$ and $MnCl_2$ each or depleted of cations by adding 20 mM EDTA and EGTA each. The lysates were precleared by three incubations each with BSA- and an irrelevant antibody-coupled Affi-gel 10 beads (Bio-Rad Laboratories, Hercules, CA) with gentle rotation at 4°C for 4 h each time. SG31 reactive membrane molecules were immunoprecipitated with SG31-coupled Affi-gel 10 beads, eluted with Laemmli sample buffer, separated by SDS-PAGE (under either reducing or non-reducing conditions), and detected by autoradiography.

In other experiments, 38B9 lymphoma cells were iodinated and lysed with 1% NP-40 lysis buffer. Precleared lysates were further depleted with three rounds of PS/2- or SG31-coupled Affi-gel 10 beads and precipitated with PS/2- and SG31-coupled Affi-gel 10 beads. Lysates, which were not depleted in this way, were precipitated with control antibody-, SG31- and PS/2-coupled beads for comparison. Eluted materials were subject to SDS-PAGE (7.5% acrylamide) under reducing conditions, and detected by autoradiography.

### Adhesion Assays

Adhesion assays were done as previously described by Hu *et al.* (1992). Briefly, Chinese hamster ovary cells (CHO) transfected with MAdCAM-1 or VCAM-1 were allowed to grow for 24 h in 96-well plates to form confluent monolayers. Alternatively, plates were coated with 1 μg/ml fibronectin (Boehringer Mannheim, Germany) in PBS for 16 h at 4°C. Subsequently, plates were washed with cell adhesion buffer (24 mM Tris–HCl [pH 7.4] containing 137 mM NaCl, 2.7 mM KCl, 2 mM glucose, 1 mM $CaCl_2$, 1 mM $MgCl_2$, and 1% BSA). Lymphoma cells were labeled for 30 min at 37°C with 12 μg/ml H33342 dye (Calbiochem, La Jolla, CA) in RPMI 1640 containing 1% BSA, washed twice with PBS, and resuspended in cell adhesion buffer supplemented with saturating amount (10 μg/ml) of control Ab, PS/2, or SG31. After incubation for 10 min at room temperature, 80,000 cells were added to each well and centrifuged for 10 min at 10*g*. Cells were allowed to adhere for 20 min at 37°C and non-adherent cells were removed by inverse centrifugation for 10 min at 50*g*. Adhesion assays were quantified by fluorimetry using a Cytofluor 2300 (Millipore, Bedford, MA). The data were represented as mean and standard deviation from three independent experiments.

### *Acknowledgements*

We thank Alexandra Lifka for technical assistance, and Ann Brookshire for the preparation of this manuscript. This work was supported NIH grant AI14782 and CA13148.

### *References*

Abitorabi, M.A., Mackay, C.R., Jerome, E.H., Osorio, O., Butcher, E.C. and Erle, D.J. (1996) “Differential expression of homing molecules on recirculating lymphocytes from sheep gut, peripheral, and lung lymph”, *J. Immunol.* **156**, 3111–3117.

Altevogt, P., Hubbe, M., Ruppert, M., Lohr, J., von Hoegen, P., Sammar, M., Andrew, D.P., McEvoy, L., Humphries, M.J. and Butcher, E.C. (1995) “The α4 integrin chain is a ligand for α4β7 and α4β1”, *J. Exp. Med.* **182**, 345–355.

Andrew, D.P., Rott, L.S., Kilshaw, P.J. and Butcher, E.C. (1996) “Distribution of alpha4 beta 7 and alpha E beta 7 integrins on thymocytes, intestinal epithelial lymphocytes and peripheral lymphocytes”, *Eur. J. Immunol.* **26**, 897–905.

Arroyo, A.G., Yang, J.T., Rayburn, H. and Hynes, R.O. (1996) “Differential requirements for alpha 4 integrins during fetal and adult hematopoiesis”, *Cell* **85**, 997–1008.

Bargatze, R.F., Jutila, M.A. and Butcher, E.C. (1995) “Distinct roles of L-selectin and integrins alpha 4 beta 7 and LFA-1 in lymphocyte homing to Peyer’s patch-HEV *in situ*: the multistep model confirmed and refined”, *Immunity* **3**, 99–108.

Bazzoni, G., Shih, D.T., Buck, C.A. and Hemler, M.E. (1995) “Monoclonal antibody 9EG7 defines a novel beta 1 integrin epitope induced by soluble ligand and manganese, but inhibited by calcium”, *J. Biol. Chem.* **270**, 25570–25577.

Berlin, C., Bargatze, R.F., Campbell, J.J., von Andrian, U.H., Szabo, M.C., Hasslen, S.R., Nelson, R.D., Berg, E.L., Erlandsen, S.L. and Butcher, E.C. (1995) “Alpha 4 integrins mediate lymphocyte attachment and rolling under physiologic flow”, *Cell* **80**, 413–422.

Brines, R. (1997) “Immune receptor supplement (Second Edition)”, *Immunol. Today* **10**, 19–34.

Cepek, K.L., Parker, C.M., Madara, J.L. and Brenner, M.B. (1993) “Integrin alpha E beta 7 mediates adhesion of T lymphocytes to epithelial cells”, *J. Immunol.* **150**, 3459–3470.

Cepek, K.L., Shaw, S.K., Parker, C.M., Russell, G.J., Morrow, J.S., Rimm, D.L. and Brenner, M.B. (1994) “Adhesion between epithelial cells and T lymphocytes mediated by E-cadherin and the alpha E beta 7 integrin”, *Nature* **372**, 190–193.

van Dinther-Janssen, A.C., Horst, E., Koopman, G., Newmann, W., Scheper, R.J., Meijer, C.J. and Pals, S.T. (1991) “The VLA-4/VCAM-1 pathway is involved in lymphocyte adhesion to endothelium in rheumatoid synovium”, *J. Immunol.* **147**, 4207–4210.

Dransfield, I., Cabanas, C., Craig, A. and Hogg, N. (1992) “Divalent cation regulation of the function of the leukocyte integrin LFA-1”, *J. Cell Biol.* **116**, 219–226.

Elices, M.J. (1994) “Leukocyte integrins”, In: Cheresh, D.A. and Mecham, R.P., eds, *Integrins, Molecular and Biological Responses to the Extracellular Matrix* (Academic Press, New York), pp. 163–194.

Engelhardt, B., Conley, F.K., Kilshaw, P.J. and Butcher, E.C. (1995) “Lymphocytes infiltrating the CNS during inflammation display a distinctive phenotype and bind to VCAM-1 but not to MAdCAM-1”, *Int. Immunol.* **7**, 481–491.

Erle, D.J., Briskin, M.J., Butcher, E.C., Garcia-Pardo, A., Lazarovits, A.I. and Tidswell, M. (1994) “Expression and function of the MAdCAM-1 receptor, integrin alpha 4 beta 7, on human leukocytes”, *J. Immunol.* **153**, 517–528.

Ferguson, T.A. and Kupper, T.S. (1993) “Antigen-independent processes in antigen-specific immunity. A role for alpha 4 integrin”, *J. Immunol.* **150**, 1172–1182.

Freedman, A.S., Munro, J.M., Rice, G.E., Bevilacqua, M.P., Morimoto, C., McIntyre, B.W., Rhynhart, K., Pober, J.S. and Nadler, L.M. (1990) “Adhesion of human B cells to germinal centers *in vitro* involves VLA-4 and INCAM-110”, *Science* **249**, 1030–1033.

Garratt, A.N. and Humphries, M.J. (1995) “Recent insights into ligand binding, activation and signalling by integrin adhesion receptors”, *Acta Anat.* **154**, 34–45.

Ginsberg, M.H., Du, X. and Plow, E.F. (1992) “Inside-out integrin signaling”, *Curr. Opin. Cell Biol.* **4**, 766–771.

Hamamura, K., Matsuda, H., Takeuchi, Y., Habu, S., Yagita, H. and Okumura, K. (1996) “A critical role of VLA-4 in erythropoiesis *in vivo*”, *Blood* **87**, 2513–2517.

Hardy, R.R., Carmack, C.E., Shinton, S.A., Kemp, J.D. and Hayakawa, K. (1991) “Resolution and characterization of pro-B and pre-pro-B cell stages in normal mouse bone marrow”, *J. Exp. Med.* **173**, 1213–1225.

Hemler, M.E. (1990) “VLA proteins in the integrin family: structures, functions, and their role on leukocytes. [Review]”, *Annu. Rev. Immunol.* **8**, 365–400.

Hu, M.C., Crowe, D.T., Weissman, I.L. and Holzmann, B. (1992) “Cloning and expression of mouse integrin beta p(beta 7): a functional role in Peyer’s patch-specific lymphocyte homing”, *Proc. Natl Acad. Sci. USA* **89**, 8254–8258.

Kilshaw, P.J. and Murant, S.J. (1990) “A new surface antigen on intraepithelial lymphocytes in the intestine”, *Eur. J. Immunol.* **20**, 2201–2207.

Koopman, G., Parmentier, H.K., Schuurman, H.J., Newman, W., Meijer, C.J. and Pals, S.T. (1991) “Adhesion of human B cells to follicular dendritic cells involves both the lymphocyte function-associated antigen 1/intercellular adhesion molecule 1 and very late antigen 4/vascular cell adhesion molecule 1 pathways", *J. Exp. Med.* **173**, 1297.

Kraal, G. (1992) "Cells in the marginal zone of the spleen", *Int. Rev. Cytol.* **132**, 31–74.

Laemmli, U.K. (1970) "Cleavage of structural proteins during the assembly of the head of bacteriophage T4", *Nature* **227**, 680–685.

Lobb, R.R. and Hemler, M.E. (1994) "The pathophysiologic role of alpha 4 integrins *in vivo*. [Review]", *J. Clin. Investig.* **94**, 1722–1728.

Masumoto, A. and Hemler, M.E. (1993) "Multiple activation states of VLA-4. Mechanistic differences between adhesion to CS1/fibronectin and to vascular cell adhesion molecule-1", *J. Biol. Chem.* **268**, 228–234.

Miyake, K., Weissman, I.L., Greenberger, J.S. and Kincade, P.W. (1991) "Evidence for a role of the integrin VLA-4 in lympho-hemopoiesis", *J. Exp. Med.* **173**, 599–607.

Moratz, C., Reese, K. and Kearney, J.F. (1994) "Characterization of B220-B cell precursors with the monoclonal antibody Ab8", *J. Cell. Biochem.* **18D**, 377, Abstract.

Oliver, A.M., Martin, F., Gartland, G.L., Carter, R.H. and Kearney, J.F. (1997) "Marginal zone B cells exhibit unique activation, proliferation and immunoglobulin secretory responses", *Eur. J. Immunol.* **27**, 2366–2374.

Picker, L.J., Treer, J.R., Nguyen, M., Terstappen, L.W.M.M., Hogg, N. and Yednock, T. (1993) "Coordinate expression of beta 1 and beta 2 integrin "activation" epitopes during T cell reponses in secondary lymphoid tissue", *Eur. J. Immunol.* **23**, 2751–2757.

Postigo, A.A., Pulido, R., Campanero, M.R., Acevedo, A., Garcia-Pardo, A., Corbi, A.L., Sanchez-Madrid, F. and de Landazuri, M.O. (1991) "Differential expression of VLA-4 integrin by resident and peripheral blood B lymphocytes. Acquisition of functionally active alpha 4 beta 1-fibronectin receptors upon B cell activation", *Eur. J. Immunol.* **21**, 2437–2445.

Pulido, R., Elices, M.J., Campanero, M.R., Osborn, L., Schiffer, S., Garcia-Pardo, A., Lobb, R., Hemler, M.E. and Sanchez-Madrid, F. (1991) "Functional evidence for three distinct and independently inhibitable adhesion activities mediated by the human integrin VLA-4. Correlation with distinct alpha 4 epitopes", *J. Biol. Chem.* **266**, 10241–10245.

Reinhardt, P.H., Elliott, J., Niu, X.F. and Kubes, P. (1996) "Functional roles for VLA-4 expressed on human neutrophils", *FASEB J.* **10**, A613, Abstract.

Ruegg, C., Postigo, A.A., Sikorski, E.E., Butcher, E.C., Pytela, R. and Erle, D.J. (1992) "Role of integrin alpha 4 beta 7/alpha 4 beta P in lymphocyte adherence to fibronectin and VCAM-1 and in homotypic cell clustering", *J. Cell Biol.* **117**, 179–189.

Sheppard, A.M., Onken, M.D., Rosen, G.D., Noakes, P.G. and Dean, D.C. (1994) "Expanding roles for alpha 4 integrin and its ligands in development", *Cell Adhesion Commun.* **2**, 27–43.

Shimizu, Y. and Mobley, J.L. (1993) "Distinct divalent cation requirements for integrin-mediated CD4 + T lymphocyte adhesion to ICAM-1, fibronectin, VCAM-1 and invasin", *J. Immunol.* **151**, 4106–4115.

St-Pierre, Y., Hugo, P., Legault, D., Tremblay, P. and Potworowski, E.F. (1996) "Modulation of integrin-mediated intercellular adhesion during the interaction of thymocytes with stromal cells expressing VLA-4 and LFA-1 ligands", *Eur. J. Immunol.* **26**, 2050–2055.

Stepp, M.A., Urry, L.A. and Hynes, R.O. (1994) "Expression of alpha 4 integrin mRNA and protein and fibronectin in the early chicken embryo", *Cell Adhesion Commun.* **2**, 359.

Stewart, M.P., Cabanas, C. and Hogg, N. (1996) "T cell adhesion to intercellular adhesion molecule-1 (ICAM-1) is controlled by cell spreading and the activation of integrin LFA-1", *J. Immunol.* **156**, 1810–1817.

Strauch, U.G., Gosslar, U., Lifka, A. and Holzmann, B. (1995) "Distinct functions of integrins alpha4beta7, alpha4beta1, and alphaIELbeta7 in lymphocyte recirculation and matrix adhesion", In: Siess, W., Lorenz, R. and Weber, P.C., eds, *Topics in Molecular Medicine* (Raven Press, New York), pp. 187–199.

Yang, J.T., Rayburn, H. and Hynes, R.O. (1995) "Cell adhesion events mediated by alpha 4 integrins are essential in placental and cardiac development", *Development* **121**, 549–560.

Yednock, T.A., Cannon, C., Vandevert, C., Goldbach, E.G., Shaw, G., Ellis, D.K., Liaw, C., Fritz, L.C. and Tanner, L.I. (1995) "Alpha 4 beta 1 integrin-dependent cell adhesion is regulated by a low affinity receptor pool that is conformationally responsive to ligand", *J. Biol. Chem.* **270**, 28740–28750.

